# Deconstructing the genetic architecture of iron deficiency chlorosis in soybean using genome-wide approaches

**DOI:** 10.1186/s12870-020-2237-5

**Published:** 2020-01-28

**Authors:** Teshale Assefa, Jiaoping Zhang, R. V. Chowda-Reddy, Adrienne N. Moran Lauter, Arti Singh, Jamie A. O’Rourke, Michelle A. Graham, Asheesh K. Singh

**Affiliations:** 1 Department of Agronomy, Iowa State University, Ames, IA USA; 2 United States Department of Agriculture, Agricultural Research Service, Corn Insects and Crop Genetics Research Unit and Department of Agronomy, Iowa State University, Ames, IA USA

**Keywords:** Gene expression, Germplasm, GWAS, GWES, Iron deficiency chlorosis (IDC), Quantitative trait locus (QTL), Soybean

## Abstract

**Background:**

Iron (Fe) is an essential micronutrient for plant growth and development. Iron deficiency chlorosis (IDC), caused by calcareous soils or high soil pH, can limit iron availability, negatively affecting soybean (*Glycine max*) yield. This study leverages genome-wide association study (GWAS) and a genome-wide epistatic study (GWES) with previous gene expression studies to identify regions of the soybean genome important in iron deficiency tolerance.

**Results:**

A GWAS and a GWES were performed using 460 diverse soybean PI lines from 27 countries, in field and hydroponic iron stress conditions, using more than 36,000 single nucleotide polymorphism (SNP) markers. Combining this approach with available RNA-sequencing data identified significant markers, genomic regions, and novel genes associated with or responding to iron deficiency. Sixty-nine genomic regions associated with IDC tolerance were identified across 19 chromosomes via the GWAS, including the major-effect quantitative trait locus (QTL) on chromosome Gm03. Cluster analysis of significant SNPs in this region deconstructed this historically prominent QTL into four distinct linkage blocks, enabling the identification of multiple candidate genes for iron chlorosis tolerance. The complementary GWES identified SNPs in this region interacting with nine other genomic regions, providing the first evidence of epistatic interactions impacting iron deficiency tolerance.

**Conclusions:**

This study demonstrates that integrating cutting edge genome wide association (GWA), genome wide epistasis (GWE), and gene expression studies is a powerful strategy to identify novel iron tolerance QTL and candidate loci from diverse germplasm. Crops, unlike model species, have undergone selection for thousands of years, constraining and/or enhancing stress responses. Leveraging genomics-enabled approaches to study these adaptations is essential for future crop improvement.

## Background

Iron (Fe) is an essential micronutrient required for multiple metabolic processes in plants including photosynthesis, respiration, and electron transport [1]. While Fe is one of the most abundant elements in the earth’s crust, aerobic conditions, high pH, and/or calcareous soils, make it insoluble and unavailable for plant use. Of the world’s cultivated soils, roughly 30% are classified as calcareous [2], including farmlands in the upper Midwestern United States where soybean is a major crop. Fe deficiency negatively affects plant growth and yield [3]. Studies in the model organism *Arabidopsis thaliana* have demonstrated that the expression of a number of underlying genes is regulated by Fe availability [4–6]. Additional genes involved in Fe acquisition and homeostasis have been the subject of numerous studies in Arabidopsis [4–9]; however, work in model species has not translated to improved Fe efficiency in crop species. Unlike Arabidopsis, which has not been domesticated, soybean was domesticated more than 5000 years ago [10]. Following domestication, soybean and other crops have been under continued selection for yield, biotic and abiotic stress tolerance, making it likely that they have developed novel strategies for dealing with Fe deficiency stress through long-term selection and mutation strategies. Therefore, a critical need exists to study Fe deficiency responses within a crop species and across a broad range of diverse genotypes.

Genome-wide association studies (GWAS) have been used to detect quantitative trait loci (QTLs) associated with important agronomic traits in rice, maize, wheat, and soybean [11–15]. Incorporating diverse plant introduction (PI) germplasm accessions in GWAS increases the likelihood of identifying novel genes and rare alleles [16]. A previous iron deficiency chlorosis (IDC) GWAS in soybean identified QTL on seven chromosomes [13]. This study was performed in two populations of advanced breeding lines developed by public and private breeding programs for the upper Midwest. However, the narrow genetic base for U.S. commercial soybean cultivars [17] limited the likelihood of identifying novel mechanisms and natural genetic variants for IDC tolerance that could be used for future soybean improvement.

GWAS usually focus on additive genetic effects. However, epistatic interactions also contribute to genetic variation [18, 19]. Detection of genome-wide epistatic (GWE) interactions represents a complementary approach to traditional genetic studies and is essential to understanding the genetic architecture of quantitative traits [20]. Studies in human and animal systems have revealed that epistatic interactions have major effects on the genetic architecture of complex disease traits [21, 22]. While the utility of GWE studies (GWES) is acknowledged in the crop research community, they have not been widely integrated.

The objective of this study was to use GWAS and GWES to examine the genetic architecture of soybean responses to Fe deficiency using a diverse panel of germplasm accessions genotyped with a dense coverage of single-nucleotide polymorphism (SNP) markers. Unlike traditional QTL or expression analyses, this combinatorial approach allowed us to identify novel genes and mechanisms conferring tolerance to IDC within the soybean germplasm collection. By coupling these studies with over 30 years of IDC research, we have deconstructed the major IDC QTL on soybean chromosome 3 (Gm03) into multiple discrete regions contributing to IDC tolerance. The identification of these candidate genes from GWAS and GWES will expand our understanding of IDC tolerance mechanisms and identify important genetic markers for soybean breeding and improvement.

## Results

### Phenotype

The phenotypic variation in IDC score among the PI accessions, averaged across replicates, ranged from highly resistant (1.0) to highly susceptible (4.7) in the 2014 field study (Additional file 1: Figure S1a) and from 1.0 to 4.6 in the 2015 field study, indicating significant differences among the PI lines for the expression of IDC symptoms (Additional file 4). The population had an approximately normal distribution for visual score and SPAD measurements at each time point. The correlation between the IDC ratings at the different time points varied from 0.71 to 0.86 in 2014 and from 0.95 to 0.98 in 2015 with the greatest correlation observed between T2 and T3 for both field and hydroponic studies (Additional file 1: Figure S1b). The estimated broad-sense heritability [23] for visual scores was 82, 64, and 52% for the first, second, and third time points, respectively. Comparing any two samples results in a positive correlation, supporting the inclusion of all collected data in our analyses.

### GWAS

A total of 97 unique SNPs were identified across all experiments, with 43, 32 and 48 unique SNPs identified from field conditions in 2014 and 2015, and hydroponics, respectively (Additional file 5, Additional file 2: Figure S2 and Additional file 3: Figure S3). In the 2014 and 2015 field data, 10 (23%) and 23 SNPs (72%) were identified in two or more time points or phenotyping methods, respectively. In the hydroponic conditions, 6 SNPs (13%) were identified in two or more time points or phenotyping methods. Twelve of the 97 total SNPs (12%) were identified across multiple years in the field data and of these, four (4%) were identified across multiple years of field data and in hydroponics. SNPs detected in more than one environment are listed in Additional file 6.

The 97 unique SNPs identified by GWAS were used to define 69 genomic regions of interest for IDC, each containing at least one significant SNP for at least one experimental measurement. On average, these regions were 35 kb in length with a standard deviation of 25.5 kb. A total of 278 genes in the Williams 82 reference genome were identified from the 69 regions of interest [24] (Additional file 7). To identify high priority candidate genes, we mined available RNA-seq data from leaves and roots of the iron efficient line Clark at 30, 60 and 120 min after iron stress [25] and at 1 and 6 h after iron stress [26]. In addition, we mined data from leaves of the iron inefficient line Isoclark 21 days post gene silencing of GmRPA3c, a previously identified and characterized iron response gene, in iron sufficient and deficient conditions [27]. Of the 278 candidate GWAS genes, 65 were significantly differentially expressed in at least one of the previous RNA-seq studies **(**Additional file 7). The majority of the differentially expressed genes (48), were identified within 2 h of iron stress application, in hydroponic conditions. Only 7 genes were identified across multiple studies, reflecting the dynamic nature of the soybean iron stress response. Of the 65 differentially expressed genes, 26 were differently expressed in leaves, 36 were differentially expressed in roots, and 5 were differentially expressed in leaves and roots.

**Fig. 1:**
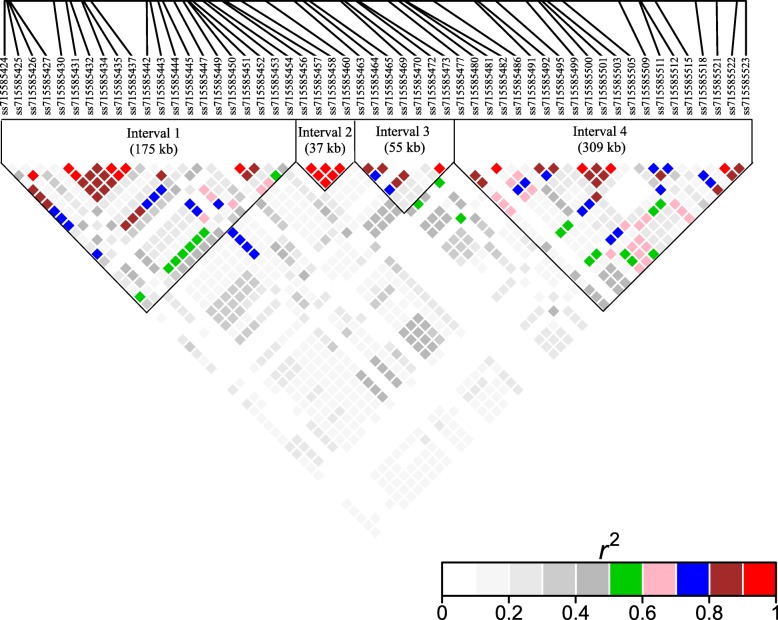
Linkage disequilibrium heat map of QTL region on chromosome 3 (Gm03). Heat map of the linkage disequilibrium (r2) of the 57 SNPs within the 576 kb region on Gm03 associated with IDC tolerance by previous QTL analyses (Lin et al., 1997; Lin et al., 1998; Peiffer et al., 2012)

Interestingly, the twelve SNPs identified across multiple years in the field data were located within a 392 kb window on Gm03 (from 34,364,354 to 35,757,151). Given that this region corresponds to a historical IDC QTL [28–31], we were interested in examining linkage across this region. Clustering (r2 > 0.5) of significant SNPs on Gm03 (34,227,914-34,955,422) identified four distinct linkage blocks (genomic intervals) within this 730 kb region (Fig. 1). Interval 1 on Gm03 spans the SNPs *ss715585424* and *ss715585454* (corresponding to genes *Glyma.03 g127900* - *Glyma.03 g129500*). Of the 17 genes in this interval, ten overlapped with the 120 kb IDC introgression identified by Peiffer et al. [31]. Five genes (*Glyma.03 g127900*, *Glyma.03 g128200*, *Glyma.03 g128300*, *Glyma.03 g129300* and *Glyma.03 g129400*) were significantly differentially expression in response to iron stress, four in roots and one in leaves (Additional file 8). Three of these have also been associated with abiotic stress and defense responses in other species (*Glyma.03 g128200*, *Glyma.03 g128300* and *Glyma.03 g129400*). Interval 2 spans SNPs *ss715585456* and *ss715585460* (corresponding to five genes: *Glyma.03 g129600* - *Glyma.03 g130000*). Within this interval both *Glyma.03 g129600* and *Glyma.03 g130000* were significantly differentially expressed in response to iron stress in roots. Interval 3 spans SNPs *ss715585463* - *ss715585473* (corresponding to six genes: *Glyma.03 g130100* - *Glyma.03 g130600*) including the final segment of the 120 kb IDC introgression reported by Peiffer et al. [31]. Of the six genes, three were differentially expressed in response to iron stress including *Glyma.03 g130200*, *Glyma.03 g130400* and *Glyma.03 g130600*. Interval 4 spans SNPs *ss715585477* - *ss715585531* (corresponding to 30 genes: *Glyma.03 g130700* to *Glyma.03 g133400*). Of these, six genes were differentially expressed in iron stress RNA-seq data including *Glyma.03 g130900*-*Glyma.03 g131200*, *Glyma.03 g132700* and *Glyma.03 g132900*.

### GWES

**Fig. 2:**
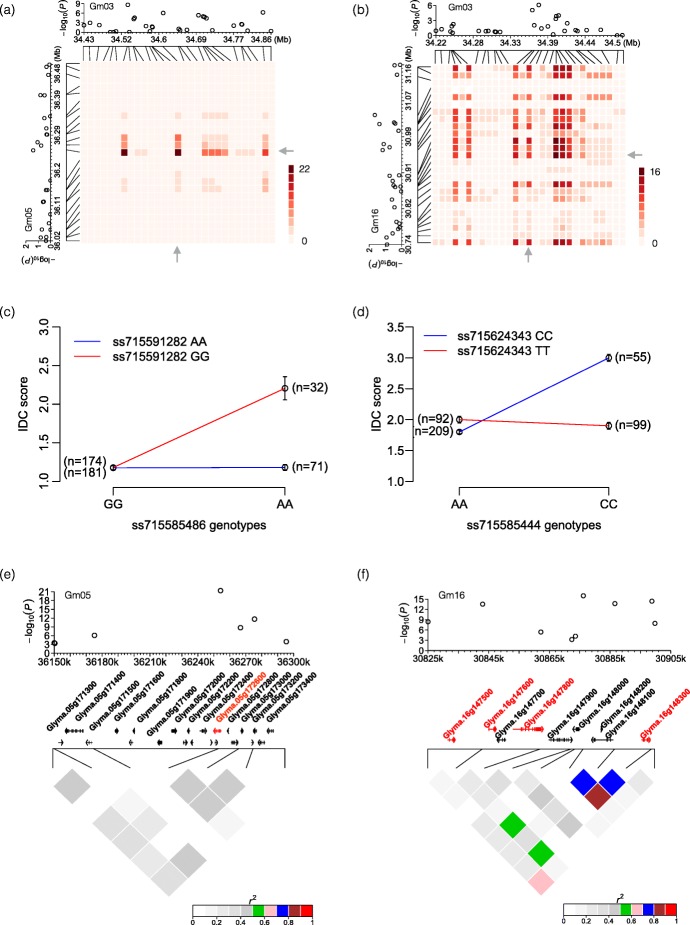
Epistatic interactions between soybean chromosome 3 (Gm03) and chromosomes 5 (Gm05) and 16 (Gm16). **a** Heat map of SNP: SNP interactions between five SNPs on Gm03 and *ss715585486* (Gm05). **b** Heat map of SNP: SNP interactions between three SNPs onGm03 and *ss715585444* (Gm16). Colors represent the -log10-transformed *P* value of each interaction. Statistically significant interactions are highlighted with arrows. **c** and **d** Impact of the AA allele of *ss715585486* (Gm05) and the TT allele of *ss715585444* (Gm16) on IDC score. Allelic combinations are provided on the X-axis with IDC visual scores on the Y-axis. These alleles confer a one-point improvement on the IDC scale. Lines that are heterozygous at the related loci were ruled out. **e** and (**f**) Candidate gene prediction for epistatic loci. The top panels show –log10 transformed *P*-values of the SNP: SNP interactions plotted in chromosomal position. Middle panels show all putative genes within the regions of interest; candidate genes of interest are highlighted in red. Linkage disequilibrium (r2) of chromosomal regions of interest for epistatic interactions are plotted in bottom panels

Epistatic tests identified 20 SNP: SNP interactions between five SNPs (*ss715585442*, *ss715585444*, *ss715585450*, *ss715585469*, and *ss715585486*) on Gm03 which interacted with 12 SNPs on other chromosomes (Fig. 2, Additional file 9). The same approach used to identify candidate genes in GWAS QTL was used to identify candidate genes within GWES regions. We identified 50 candidate genes (Additional file 10 and Additional file 11), of which 13 were differentially expressed in the different iron stress responsive RNA-seq data sets. All five SNPs on Gm03 exhibited significant interactions with *ss715591282* on Gm05 (Fig. 2a, c and e). The SNP is not located within any previously identified IDC QTL. The AA allele of this SNP confers a one point improvement on the IDC scale compared to the GG allele (Fig. 2c). Three of the five SNPs from Gm03 interact with *ss715624343* (Fig. 2b, d and f); this SNP lies on soybean Gm16, which has not previously been reported as an IDC QTL. Analysis shows that a TT allele of *ss715624343* accounts for a one-point increase on the five-point chlorosis rating scale compared to the CC allele.

In order to validate GWES results, we took advantage of StringDB, which stores known interactions between proteins [32]. This confirmed an interaction between candidate gene *Glyma.03 g129400* (AtBIGYIN), which responds to iron stress (Additional file 7), and *Glyma.16 g148100* (At5g55610, Additional file 11). In Arabidopsis, both of these proteins are located within the outer mitochondrial membrane and are thought to a play a role in mitochondrial signaling during stress [33].

## Discussion

### Historical discovery of the IDC QTL on soybean Gm03

**Fig. 3:**
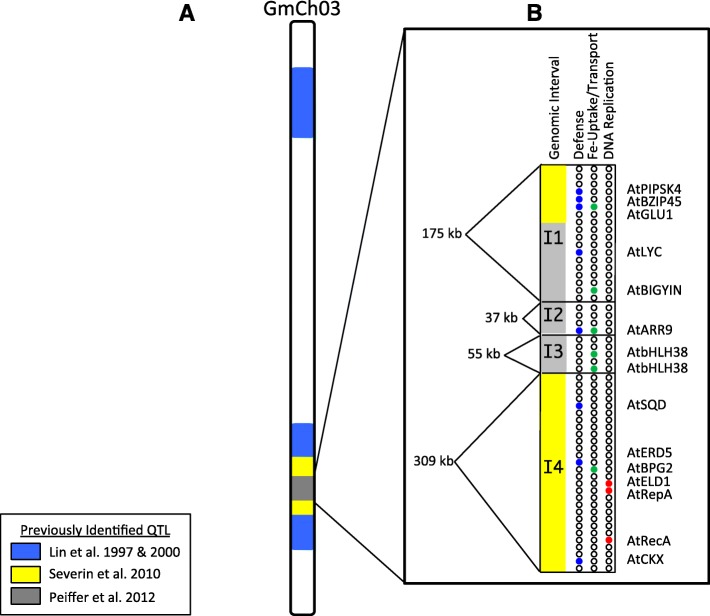
Deconstructing the IDC QTL on soybean chromosome 3 (Gm03) reveals multiple genes provide IDC tolerance. **a**) Lin et al. (Lin et al., 1997; Lin et al., 2000) identified a major QTL on Gm03 responsible for > 70% phenotypic variation in IDC tolerance (shown in blue). Severin et al. (Severin et al., 2010); identified the introgressed region (shown yellow). Peiffer et al. (Peiffer et al., 2012) used fine mapping of sub-NILs to further fine map the QTL (shown in grey). **b** Linkage disequilibrium analysis of the 57 SNPs spanning a 730 kb within the original Lin et al. (Lin et al., 1997) QTL divided this region into four distinct genomic intervals (I1, I2, I3, and I4). Each interval contains high priority candidate genes of interest (I1 = 5 genes; I2 = 1gene, I3 = 2 genes, I4 = 7 genes) that may be involved in conferring Fe deficiency tolerance either through iron-stress responsive (pink), enhanced defense (blue), Fe uptake/transport (green), or altered DNA replication (red). Additional details on this region are provided in Additional file 8)

Over the last 35 years, several different approaches have been used to identify genes conferring tolerance to IDC in soybean. Cianzio et al. [34] and Cianzio and Fehr [35] were the first to demonstrate the genetic inheritance of IDC tolerance in soybean. In 1997 and 1998, Lin et al. [28, 36] used field and hydroponic studies to identify an IDC QTL on soybean Gm03 that explained 70% of the phenotypic variation (Fig. 3). In 2010, Severin et al. [30] used next-generation sequencing data to identify the introgression from Fe inefficient T203 into iron-efficient Clark, used to develop inefficient Isoclark. Peiffer et al. [31] fine mapped the introgressed region, using Clark and Isoclark sub-NILs to identify a 120 kb region conferring IDC tolerance. Of the eighteen genes in this region, two candidate genes in soybean were identified as having the greatest homology to the AtbHLH38 transcription factor in Arabidopsis that regulates Fe uptake in the root [37].

### Expression studies of IDC tolerance in soybean

While mapping studies suggested that a single gene likely controlled IDC response in Clark, expression studies suggested the involvement of multiple regulatory cascades. In 2009, O’Rourke et al. [38] compared gene expression between Clark and Isoclark leaves following Fe stress treatment. This study suggested that Isoclark contained a mutation responsible for regulating the expression of downstream Fe uptake and transport genes. It also suggested that regulation of DNA replication and defense was an important component of Clark’s Fe stress response. Atwood et al. [27] used virus-induced gene silencing (VIGS) to repress the expression of GmRPA3c (Replication Protein A, subunit 3c) in Isoclark to mirror the expression observed in Clark. GmRPA3c silencing improved Isoclark performance during Fe stress and resulted in massive transcriptional reprogramming of genes involved in iron uptake and homestasis, defense/cell death, DNA replication, abiotic stress, regulation of the circadian clock, and autophagy. Differential expression of DNA replication genes has now been observed as early as one hour after Fe stress treatment in soybean [26]. The involvement of DNA replication in abiotic and biotic stress responses has now been reported in multiple crop species including maize, barley, and onion [39–43].

The differential expression of Fe, defense, and DNA replication cascades in soybean suggest the involvement of at least two regulatory genes in soybean’s Fe stress response. Peiffer et al. [31] identified two candidate BHLH38 transcription factors in the IDC QTL on Gm03 induced by Fe stress in roots. Multiple constructs have been developed and used to silence these genes, but silenced Clark plants had no significant phenotypic changes when grown under Fe stress conditions. While negative results do not preclude the involvement of these soybean BHLH38 genes in Clark’s Fe stress response, it again suggests the involvement of additional genes. Further, there is no evidence that AtBHLH38 regulates the expression of DNA replication or defense genes, nor that regulation of these genes is a component of the Arabidopsis Fe stress response [6, 44, 45]. Therefore, it is likely that regulation of the DNA replication and defense machinery, in response to abiotic stress, is a unique adaptation in crop species. Since only one major IDC QTL has been identified in soybean, it would suggest that multiple candidate genes reside within this region.

### Splitting the IDC QTL on soybeanGm03

Of the 69 genomic regions identified in this study, eight overlapped with the previously identified IDC QTL [28, 29] and introgressed regions [30] on Gm03. Within this region, 13 of 16 significant and unique SNPs were identified across three or more experimental conditions. Given the physical distribution of the SNPs across this 730 kb region, the linkage disequilibrium (LD) between these SNPs was examined. This approach broke the previously described IDC QTL into four distinct intervals of 175, 37, 55 and 309 kb (intervals 1–4, respectively, Figs. 1 and 3), demonstrating that multiple genes within this window on Gm03 contribute to IDC tolerance. Intervals 2 and 3 completely overlapped with the 120 kb introgression identified in a previous study [31]. In contrast, interval 4 did not overlap with the Peiffer IDC introgression at all. Within each of the intervals, high priority candidate genes involved in signal transduction were identified that could explain the hallmarks of soybean’s Fe stress response: defense, DNA replication, and Fe uptake/transport.

Interval 1 contained four high priority candidate genes of interest: *Glyma.03 g128200* (AtTGA6), *Glyma.03 g128300* (AtGLU1), *Glyma.03 g128900* (AtLYC), and *Glyma.03 g19400* (AtBIGYIN). AtTGA6 regulates cross-talk between salicylic acid (SA) and jasmonic acid (JA)/ethylene defense responses [46]. All three hormones are also involved in the regulation of Fe deficiency responses [47, 48]. AtGLU1 encodes a ferredoxin-dependent glutamate synthase. AtGLU1 knock-downs are slightly cholorotic [49]. Gene expression analyses of AtGLU1 reveal extensive transcriptional reprogramming including repression of photosynthesis-related genes and induction of abiotic stress-associated genes. Similarly, AtLYC is involved in non-photochemical quenching under high light conditions [50]. Transformation of AtLYC in tobacco resulted in increased tolerance to salt stress. AtBIGYIN regulates mitochondrial size and number [51]. Mitochondria are essential for the synthesis of Fe-S clusters, which are linked to intracellular Fe homeostasis [52]. All of these genes, except *Glyma.03 g128900* (AtLYC), were differentially expressed in roots 30 min after iron stress (Additional file 8).

Interval 2 contained a single high priority candidate gene. *Glyma.03 g130000* was differentially expressed in response to Fe stress in the roots at 30 min (Additional file 8). *Glyma.03 g130000* encodes a homolog of AtRR4 (Response Regulator 4), a component of the cytokinin-signaling pathway. Cytokinin signaling regulates a broad range of nutrient and environmental stress responses [53] and is directly or indirectly involved in regulating Fe uptake genes [54]. AtARR4 is also directly involved in the regulation of the circadian clock and the cell cycle. The circadian clock in turn regulates the Fe homeostasis genes AtIRT1, AtBHLH38, and AtFERRITIN1 [55]. Similarly, Atwood et al. [27] and Moran Lauter et al. [26] observed differential expression of circadian clock genes in response to Fe stress in soybean.

Interval 3 contained the two AtBHLH38 (*Glyma.03 g130400* and *Glyma.03 g130600*) transcription factors characterized by Peiffer et al. [31]. AtBHLH38 interacts with FIT and directly enhances the expression of downstream Fe genes FRO2 and IRTI [37]. Both genes are differentially expressed in response to Fe in the roots at 30 min (Additional file 8). *Glyma.03 g130600* was also differentially expressed in the roots at 1 h (Additional file 8). Soybean lines containing a 12 bp deletion in *Glyma.03 g130400* were unable to induce the expression of the Fe uptake genes GmFRO2 and GmFIT1 under Fe stress conditions.

Interval 4 is likely one of the most novel regions because our analyses placed it outside the 120 kb introgression identified by Peiffer et al. [31]. This region contains four high priority candidate genes: *Glyma.03 g130900* (AtSDP1), *Glyma.03 g131100* (AtSQD1), *Glyma.03 g132400* (At1G52950) and Glyma.03 g133000 (AtRECA). Homologs of SDP1 play an essential role in lipid metabolism and cell survival during stress conditions in yeasts, mammals and plants [56]. While AtSQD1 mutants have no obvious phenotypes, AtSQD2 mutants exhibit severe chlorosis under phosphate starvation conditions [57]. Both *Glyma.03 g130900* and *Glyma.03 g131100* were repressed by iron stress in leaves at 120 min. *Gly*ma.03 g131100 was induced by iron stress in roots at 30 min (Additional file 8). *Glyma.03 g132400* is homologous to RPA subunit 1 and contains the RPA1 domain (PTHR23273). RPA is a heterotrimeric protein made of three subunits (RPA1, RPA2 and RPA3) which bind single-stranded DNA during DNA repair and replication [58]. Atwood et al. [27] demonstrated that the majority of RPA subunits respond to Fe stress in soybean. Further, silencing of RPA subunit 3c (GmRPA3c) restored IDC tolerance in Isoclark. AtRECA regulates DNA repair through homologous recombination and is also an essential component of DNA replication [59]. Since RECA is targeted to the choloroplast, loss of RECA results in leaf abnormalities associated with the lack of DNA repair. RPA and AtRECA are both important components of the DNA replication and repair machinery (KEGG: ath03030, ath03420, ath03430, athh03440). Biotic and abiotic stresses, like IDC, result in the release of reactive oxygen species that can damage DNA [43]. Recognition of DNA damage results in inhibition of cell proliferation, allowing repair to occur. This also inhibits growth, reducing the need for iron.

### GWAS identifies novel IDC genes throughout the soybean genome

Population-based association studies involving unrelated individuals are promising approaches for identifying the genetic basis of complex traits such as IDC tolerance. Previous soybean IDC studies were limited by their reliance on homology to IDC-associated genes in a model species or on differential gene expression in response to Fe stress in a limited number of genotypes, conditions, or tissues. Using multiple phenotyping methods, developmental stages, growth conditions, and a diverse germplasm panel, would allow us to discover multiple mechanisms governing IDC tolerance in the soybean germplasm collection.

SNPs significantly associated with Fe deficiency were identified on every chromosome except Gm04. The genomic regions on Gm03 and Gm19 reside within previously identified Fe efficiency QTL [28, 60, 61]. The remaining significant SNPs and the associated 228 genes identified throughout the genome were unique to this study. Fe specific stress response genes included genes involved in heavy metal sensing, uptake, and homeostasis. AtURH2 (*Glyma.20 g000200*) is involved in Fe sensing in yeast and mammals [62], and expression of AtOSX3 (*Glyma.06 g056600*) confers tolerance to high metal levels and oxidizing chemicals [63]. *Glyma.08 g347000* is homologous to AtMRP3, a multi-drug resistance-associated protein induced by multiple heavy metals, but not Fe [64].

Disease and stress-related genes associated with significant SNPs had functions conferring tolerance to various stresses including heat stress (*Glyma.05 g001200* and *Glyma.06G056400* [48, 64, 65]), cold stress (*Glyma.05G000200* and *Glyma.12G235700* [48, 65, 66]), cadmium tolerance (*Glyma.09G110400*, [64]), shade avoidance (*Glyma.14G032200*, [67]), salt stress (*Glyma.09G051200*, [68]), and phosphate deficiency (*Glyma.09G110200*, [69]). Genes contributing to disease and pathogen responses included four genes on Gm19 encoding canonical disease resistance genes (*Glyma.19G139400*, *Glyma.19G139500*, *Glyma.19G139600*, and *Glyma.19G139700*), though none have been associated with specific diseases. Additional disease response genes include the homologs to AtCDR1 (*Glyma.12G235400*), a constitutive disease response gene involved in Pseudomonas syringae responses, AtPCC1 (*Glyma.15G256200* and *Glyma.15G256300*) which is involved in RPP7-dependent resistance to downy mildew [70–72], and AtLECRK (*Glyma.02 g043000*), involved in resistance to Phytophthora *spp.*

DNA replication and cell growth associated genes with significant SNPs included *Glyma.01G193400* (AtCYCD5), *Glyma.02G074800* (AtGAI), and *Glyma.05G168600* (AtEBS1), all important components regulating plant growth and the cell cycle [73–75]. In addition, significant SNPs were also identified in two sucrose transporter (AtSUC2) homologs (*Glyma.02 g075000* and *Glyma.02 g075100*). Increased sugar accumulation in roots induces reductase activity and the expression of Fe acquisition genes [76]. A family of SWEET sugar transporters and two SUC2 genes were differentially expressed after 1 h of Fe deficiency stress in soybean, confirming the importance of sugar signaling in soybean’s Fe stress response [26]. The identification of genes involved in Fe specific, general biotic/abiotic stress responses and DNA replication in this experiment confirms that Fe deficiency responses in soybean occur through multiple novel mechanisms.

### Iron deficiency and epistasis

Epistatic interaction analyses identified five SNPs on Gm03 that fell within three of the four genomic intervals depicted in Fig. 3 (three SNPs in interval one, one SNP each in interval three and four). Cumulatively, these 5 SNPS had epistatic interactions with 13 different genomic locations, representing 50 candidate interacting genes. Each of these SNPs interacts with SNP *ss715591282*, which is located on Gm05 (Fig. 2a, Additional file 9). This SNP is not located within any previously identified IDC QTL (SoyBase.org). Further, it is not associated with any QTL identified in this GWAS study. The AA allele of this SNP confers a more than one point improvement on the IDC scale compared to the GG allele (Fig. 2c). A one-point increase on a five-point IDC rating scale corresponds to a 20% reduction in yield [3]. Genomic analyses of the SNP identified nine candidate genes, three of which were differentially expressed in response to iron stress. *Glyma.05 g172300* (AtPSBY) encodes a component of photosystem II, which produces reactive oxygen species in response to stress [77]. The function of the other two iron responsive genes (*Glyma.05 g172400* and *Glyma.05 g172800*) is unknown. It is worth noting the SNP itself is located within *Glyma.05 g172600* (Fig. 2e). The Arabidopsis homolog of this gene is Actin Regulated Protein 3 (AtARP3), involved in regulating Ca2+ signaling in response to salt stress [78]. Though calcium signaling has not yet been characterized in IDC responses, it is a conserved signaling response known to be involved in multiple nutrient deficiencies including phosphate, potassium, boron, and salt. It is possible that any of these nine genes in this region are involved in the epistatic interaction associated with SNP *ss715591282*.

**Fig. 4:**
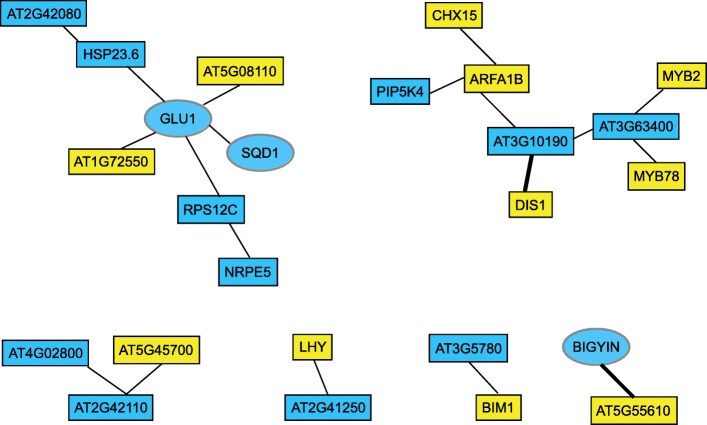
Identification of potential gene interactions from GWAS and GWES. Five GWAS SNPs from Gm03 had epistatic interactions with 12 SNPs through the soybean genome. Given the complexity of Gm03 region, all Gm03 candidate genes were tested for potential interactions with all candidate GWES genes using StringDB (REF). This identified six potential GWAS-GWES interaction networks. Candidate GWAS genes are in blue and candidate GWES genes are in yellow. Genes that are differentially expressed in response to iron stress are in ovals. Bold lines confirm predicted GWAS SNP and GWES SNP interactions

An additional epistatic interaction involves three SNPs on interval one that interact with SNP *ss715624343* on Gm16 (Fig. 2b). No IDC QTL have been previously discovered on Gm16 (SoyBase.org). While three candidate genes were identified in this region, none were differentially expressed in response to iron stress or had obvious function related to biotic or abiotic stress responses. Remarkably, the TT allele of this SNP confers a > one point improvement on the IDC scale compared to the CC allele (Fig. 2d). Within the GWAS candidate genes for SNP *ss715585450*, we identified *Glyma.03 g129400* (AtBIGYIN), which responded to iron stress. Using StringDB [32], we were able to identify a candidate epistatic gene corresponding to SNP *ss715624343*, *Glyma.16 g148100*. Given the complexity of the Gm03 regions and that three distinct non-consecutive GWAS SNPs were able to interact with multiple genomic locations, we decided to use StringDB to test the interactions of all Gm03 candidate GWAS genes with all the candidate GWES genes. This identified six potential networks, each containing at least one GWAS and GWES candidate gene (Fig. 4). Of the six networks, two contained iron stress responsive genes. Of particular interest was the network containing the GWAS iron stress responsive gene *Glyma.03 g128300* (AtGLU1). AtGLU1 encodes a ferredoxin-dependent glutamate synthase. Knock-down mutants display leaf chlorosis and activation of multiple stress responses [49]. In the network, AtGLU1 interacts with the GWES candidate genes *Glyma.05 g127900* (At1G72550), a tRNA synthase, and *Glyma.06 g206600* (At5G08110/AtHRQ1), which is required for genome stability and repair [79]. These results provide further support for our epistatic analyses. Exploiting these novel findings could result in improved crop performance under stress conditions.

## Conclusions and perspectives

In this report, we identified a significant number of molecular markers, genomic regions and candidate genes responding to iron deficiency. Leveraging genomics-enabled approaches to study iron deficiency chlorosis is essential for future soybean improvement programsunder multiple objectives [80]. Genome wide studies will benefit from digital and automated phenotyping [81, 82, 83]. Also, markers reported in this study might help in future soybean genomic studies. Our genome-wide studies leveraged thousands of SNPs, a diverse soybean germplasm panel, multiple phenotyping methods, developmental stages and growth conditions. This approach allowed us to identify novel IDC QTL that could be used for future soybean improvement. By integrating gene expression data from RNA-seq studies of soybean iron stress responses, we were able to identify high priority candidate genes. The novel GWES study allowed us to identify novel gene networks contributing to iron stress responses in soybean. In addition, we were able to dissect the historical IDC QTL on Gm03 into four distinct genomic intervals. For more than 30 years, it was thought that a single candidate gene controlled this QTL accounting for as much as 70% of the observed phenotypic variation in Fe deficiency tolerance. Only through an interdisciplinary approach that combined 30 years of breeding, gene expression, and new genome-wide association studies could we demonstrate that this region contains multiple candidate genes. Linkages to the same biochemical and molecular pathways suggest there are multiple avenues for generating tolerance to IDC in soybean that can be leveraged for future crop improvement.

## Methods

### Plant materials

This study included 460 soybean PI accessions from 27 countries obtained from the USDA National Plant Germplasm System (www.ars-grin.gov, Additional file 4). Accessions were classified by maturity group with 31, 36, and 33% classified as maturity groups I, II, and III, respectively. A randomized complete block design was used with two replicates in 2014 and four replicates in 2015. In 2014, three to four seeds of each genotype were hand planted in 0.3 m long plots with a 0.91 m plot-to-plot distance (alleyway) and 0.76 row to row spacing. In 2015, five seeds were hand planted in 0.3 m long hill plots with 0.61 m plot to plot distance (alleyway) and 0.76 m row to row spacing. Weeds that emerged after sowing were controlled by hand-weeding.

The PI accessions were also evaluated under hydroponic conditions at the Iowa State University Agronomy Department greenhouse in 2015 under 16-h photoperiods. Five seeds of each PI accession were germinated on germination paper for 7 days. Uniform seedlings of each accession were then transplanted into a hydroponic system containing 240 L of an Fe deficient medium supplemented with a daily nutrient solution as described by Chaney et al. [84]. The experiment was set up as a randomized complete block design with two replicates. Soybean IDC checks Clark (IDC tolerant) and Isoclark (IDC susceptible) were included.

### Experimental field and soil testing

An experimental plot previously used for IDC studies was selected for the field experiments at the Bruner farm, Iowa State University. Soil sampling was conducted using a soil probe (JMC Soil Samplers, Newton, IA) and each sample was analyzed at the Soil and Plant Analysis Laboratory at Iowa State University. The soil parameters measured were pH (1:1, H2O: soil) [85], calcium carbonate content [86], and Fe content [87].

### Iron deficiency chlorosis evaluation

In the field, IDC symptoms were rated on a visual scale of 1 (no chlorosis) to 5 (severe chlorosis, stunting, and necrosis) at T1 (representing V2 to V3), T2 (representing V5 to V6), and T3 (representing R1, approximately 2 weeks after T2) (Additional file 1: Figure S1a) [34, 88]. Chlorophyll concentration was measured using a Soil Plant Analysis Development (SPAD) meter in the field at T1 and T2. In the hydroponic system, visual phenotypes were recorded at T1, T2 and T3 representing the V1, V2 and V3 trifoliate stages, respectively. SPAD measurements were taken at V1 and V2.

### Genotyping and quality control

All 460 PI accessions were genotyped using the Illumina Infinium SoySNP50K BeadChip as described in previous studies [89], and data was obtained from SoyBase (https://soybase.org/snps/) which contains 42,506 high confidence SNPs. BEAGLE genetic analysis software (version 3.3.1, [90]) was used with the default settings to impute missing data. Markers missing at a frequency greater than 10% were removed from further analyses. After imputation, SNPs with a minor allele frequency less than 5% were removed from the data set. A total of 36,139 SNPs was used for GWA and GWE analyses.

### Linkage disequilibrium

The LD between markers was calculated as the squared allelic frequency correlation coefficient (r2) using the R package synbreed [91]. The r2 value was calculated independently for euchromatic and heterochromatic regions because of significant differences in the recombination rates between the two regions [92]. The physical lengths of euchromatin and heterochromatin for each chromosome were determined from SoyBase (https://soybase.org/SequenceIntro.php, version Williams82.a2. v1, [24]). The r2 values for SNPs with a pairwise distance less than 10 Mbp in either euchromatic or heterochromatic regions were plotted on a LD decay graph [93] using R [94]. The rate for the LD decay was determined as the chromosomal distance at the point where the average r2 dropped by half [95].

### GWAS and GWES

The IDC phenotypic data for each PI accession at each time point were analyzed using the best linear unbiased prediction (BLUP) and the R package lme4 [96] to reduce the effects of environmental variation. The mixed linear model accounting for familial relationship was fitted for each time point by using the genome association and prediction integrated tool (GAPIT) R package [97, 98]. Using the Bayesian information criterion test of model fitness, no population structure was detected likely due to the use of genetically diverse core collection genotypes in this study (Additional file 12, [99, 100]). The empirical significance level *P* < 0.001, determined by 1000 permutations, was used as the threshold for SNPs-trait associations [92]. Considering the low decay rate of the genome-wide LD in soybean [92], we used LD r2 > 0.5 with the most significant SNP to cluster nearby SNPs to form the QTL, and the most significant SNP was picked to represent each locus, which resulted in multiple loci at the previously reported major-effect locus on Gm03. The genome-wide epistatic interactions between SNP pairs were analyzed using the software PLINK version 1.07 [101, 102]. To correct the multiple comparisons of SNPs, a Bonferroni threshold of α = 0.05 was used [103].

### Prediction of candidate genes

To identify the GWAS and GWES genomic intervals containing candidate genes, the two closest non-significant SNPs on either side of a significant SNP were identified (Additional file 5). The non-significant SNPs were then queried against the SoyBase genome browser (www.soybase.org/gb2/gbrowse/gmax2.0/) to determine their position in the genome and to identify all genes located between SNPs. Overlapping SNP intervals were combined into a single genomic region. The identified genes were then annotated using the SoyBase annotation tool (www.soybase.org/genomeannotation/), which provided the best Arabidopsis homolog (The Arabidopsis Information Resource version 10, www. arabidopsis.org). Literature searches for Arabidopsis homologs were used to identify genes with functions related to Fe deficiency and abiotic stress tolerance. The soybean genes located within a SNP interval were also queried against other soybean Fe deficiency genes reported in previous publications, to link candidate genes with genes differentially expressed in response to IDC and previously reported IDC QTL [15, 26–28, 30, 31, 36, 38, 61]. When necessary, the SoyBase Gene Model Correspondence Lookup (https://www.soybase.org/correspondence/) was used to compare gene expression data from different genome assemblies. To examine potential candidate gene interactions, the Arabidopsis homologs of candidate genes from all Gm03 GWAS and all GWES were examined using StringDB version 10.5 [28].

### Statistical analysis

The model for IDC visual scores and SPAD measurement data collected at each time point was yijk = μ + gi + bj + eijk, where μ is the total mean, gi is the genetic effect of the ith genotype, bj is the block effect, and eijk is the residual effect including random error and possible interaction between genotype and block. Broad-sense heritability estimates for IDC were calculated on an entry mean basis using the equation, H^2^ = σ^2^_g_ / [σ^2^_g_ + (σ^2^_g*y_/y) + (σ^2^_e_/ry)], where σ^2^_g_ = genotypic variance, σ^2^_g*y_ = genotype by year interaction, y = number of years, and *r* = number of replicates [21]. Estimation of variance components was computed using SAS version 9.3 (SAS Institute Inc., Cary, NC).

## Supplementary information

**Additional file 1: Figure S1.** (a and b). Field chlorosis ratings and correlations across treatments and time.

**Additional file 2: Figure S2.** Manhattan plots of IDC GWAS for each time point.

**Additional file 3: Figure S3.** Venn diagrams illustrating the distribution of significant SNPs associated with IDC detected at different time points.

**Additional file 4.** Soybean Plant Introduction (PI) names, maturity group, and IDC scores for lines utilized in GWAS and GWES analyses.

**Additional file 5.** Identification of genomic regions containing SNPs significantly associated with IDC.

**Additional file 6.** Statistics of the peak SNPs of the QTL associated with soybean IDC tolerance across environments.

**Additional file 7.** Characterization of candidate genes in GWAS QTL.

**Additional file 8.** Characterization of candidate genes in the historical IDC QTL on soybean Gm03.

**Additional file 9.** Significantly associated epistatic (SNP-SNP) interactions for iron deficiency chlorosis.

**Additional file 10.** Identification of genomic regions containing epistatic SNPs (GWES) associated with IDC.

**Additional file 11.** Characterization of candidate genes in GWES regions.

**Additional file 12.** Bayesian Information Criterion values of mixed linear model with principal components (PCs) applied for association analysis of iron deficiency chlorosis in soybean population.

## Data Availability

The datasets supporting the conclusions of the present study are included within this article (and its additional files). Data not included in the supplemental files can be obtained by request to the corresponding author. Seed used in experiments can be obtained from the USDA National Plant Germplasm System.

## Abbreviations

BLUP: Best linear unbiased prediction
GWAS: Genome-wide association study
GWE: Genome wide epistasis
GWES: Genome wide epistatic study
IDC: Iron deficiency chlorosis
LD: Linkage disequilibrium
QTL: Quantitative trait loci
SNP: Single nucleotide polymorphism
