# Supplementary material for: Deconstructing the genetic architecture of iron deficiency chlorosis in soybean using genome-wide approaches
Source: BMC Plant Biol. 2020 Jan 28;20:42. doi: 10.1186/s12870-020-2237-5 (PMC6988307; doi:10.1186/s12870-020-2237-5)
Supplement: Supplementary file 1 — Additional file 1: Figure S1. (a and b). Field chlorosis ratings and correlations across treatments and time. [file 12870_2020_2237_MOESM1_ESM.docx]

**a**

**b**

**Additional file 1: Figure S1. Field chlorosis ratings and correlations across treatments and time. (a)** IDC symptoms were rated on a visual scale of 1 (no chlorosis) to 5 (severe chlorosis and necrosis). Panel demonstrates responses at T2 (V5 to V6 stage). (**b)** Chlorosis scores were assigned at T1 (V2 to V3 stage), T2 (V5 to V6 stage), and T3 (R1 stage, two weeks after the T2 collection) in 2014 and 2015. Similarly, in the hydroponic system (HD), visual phenotypes were recorded at T1, T2 and T3 representing the V1, V2 and V3 trifoliate stages, respectively. Pearson’s correlation of IDC scores between evaluation time points and conditions.
