# Supplementary material for: Deconstructing the genetic architecture of iron deficiency chlorosis in soybean using genome-wide approaches
Source: BMC Plant Biol. 2020 Jan 28;20:42. doi: 10.1186/s12870-020-2237-5 (PMC6988307; doi:10.1186/s12870-020-2237-5)
Supplement: Supplementary file 2 — Additional file 2: Figure S2. Manhattan plots of IDC GWAS for each time point. [file 12870_2020_2237_MOESM2_ESM.docx]

**Additional file 2: Figure S2**. **Manhattan plots of IDC GWAS for each time point.** Evaluation of IDC in field grown plants at T1, T2, and T3 in 2014 (a) and 2015 (b). (c). IDC evaluation in hydroponic grown plants at T1 and T2. The horizontal black line shows the genome-wide significance threshold (FDR < 0.05). Visual IDC scores were assigned at different growth stages: T1 (V2-V3), T2 (V5-V6) and T3 (R1, two weeks after T2 measurements). HD refers to hydroponic growth conditions.
