# Supplementary material for: Deconstructing the genetic architecture of iron deficiency chlorosis in soybean using genome-wide approaches
Source: BMC Plant Biol. 2020 Jan 28;20:42. doi: 10.1186/s12870-020-2237-5 (PMC6988307; doi:10.1186/s12870-020-2237-5)
Supplement: Supplementary file 3 — Additional file 3: Figure S3. Venn diagrams illustrating the distribution of significant SNPs associated with IDC detected at different time points. [file 12870_2020_2237_MOESM3_ESM.docx]

**Additional file 3: Figure S3. Venn diagrams illustrating the distribution of significant SNPs associated with IDC detected at different time points**. **A** and **B.** Significant SNPs identified in in 2014 (A) and 2015 (B) field evaluations. **C.** Distribution of significant SNPs identified at T1 and T2 in plants grown in hydroponic conditions. Time points are associated with distinct soybean developmental stages: : T1 (V2-V3), T2 (V5-V6) and T3 (R1, two weeks after T2 measurements). SPAD measurements were taken at V1 (SPAD1) and V2 (SPAD2).
